# Insect chemical ecology: chemically mediated interactions and novel applications in agriculture

**DOI:** 10.1007/s11829-020-09791-4

**Published:** 2020-11-09

**Authors:** Crispus M. Mbaluto, Pascal M. Ayelo, Alexandra G. Duffy, Anna L. Erdei, Anaїs K. Tallon, Siyang Xia, Gabriela Caballero-Vidal, Urban Spitaler, Magdolna O. Szelényi, Gonçalo A. Duarte, William B. Walker, Paul G. Becher

**Affiliations:** 1 Molecular Interaction Ecology, German Centre for Integrative Biodiversity Research (iDiv) Halle-Jena-Leipzig, Pusch straße 4, 04103 Leipzig, Germany; 2 Institute of Biodiversity, Friedrich-Schiller-Universität Jena, Dornburger Str. 159, 07743 Jena, Germany; 3 International Centre of Insect Physiology and Ecology (Icipe), P.O. Box 30772-00100, Nairobi, Kenya; 4 Department of Zoology and Entomology, University of Pretoria, Hatfield, Private Bag X20, Pretoria, 0028 South Africa; 5 Evolutionary Ecology Laboratories, Department of Biology, Brigham Young University, 4102 Life Science Building, Provo, UT 84602 USA; 6 Zoology Department, Plant Protection Institute, Centre for Agricultural Research, Herman Ottó str. 15, Budapest, 1022 Hungary; 7 Department of Plant Protection Biology, Swedish University of Agricultural Sciences, P.O. Box 102, 23053 Alnarp, Sweden; 8 Department of Ecology and Evolutionary Biology, Yale University, 21 Sachem Street, New Haven, CT 06511 USA; 9 INRAE, Institute of Ecology and Environmental Sciences of Paris, CNRS, IRD, UPEC, Sorbonne Université, Université Paris Diderot, Route de Saint-Cyr, 78026 Versailles Cedex, France; 10 Institute of Plant Health, Laimburg Research Centre, Laimburg 6, 3904 Ora, South Tyrol Italy; 11 Department of Crop Sciences, Institute of Plant Protection, University of Natural Resources and Life Sciences (BOKU), Gregor-Mendel-Straße 33, 1180 Vienna, Austria; 12 LEAF-Linking Landscape, Environment, Agriculture and Food Instituto Superior de Agronomia, Universidade de Lisboa, Tapada da Ajuda, 1349-017 Lisbon, Portugal

**Keywords:** Chemical ecology, Trophic interactions, Plant defenses, Insect olfaction, Semiochemicals, Integrated pest management, Remote teaching

## Abstract

Insect chemical ecology (ICE) evolved as a discipline concerned with plant–insect interactions, and also with a strong focus on intraspecific pheromone-mediated communication. Progress in this field has rendered a more complete picture of how insects exploit chemical information in their surroundings in order to survive and navigate their world successfully. Simultaneously, this progress has prompted new research questions about the evolution of insect chemosensation and related ecological adaptations, molecular mechanisms that mediate commonly observed behaviors, and the consequences of chemically mediated interactions in different ecosystems. Themed meetings, workshops, and summer schools are ideal platforms for discussing scientific advancements as well as identifying gaps and challenges within the discipline. From the 11th to the 22nd of June 2018, the 11th annual PhD course in ICE was held at the Swedish University of Agricultural Sciences (SLU) Alnarp, Sweden. The course was made up of 35 student participants from 22 nationalities (Fig. 1a) as well as 32 lecturers. Lectures and laboratory demonstrations were supported by literature seminars, and four broad research areas were covered: (1) multitrophic interactions and plant defenses, (2) chemical communication focusing on odor sensing, processing, and behavior, (3) disease vectors, and (4) applied aspects of basic ICE research in agriculture. This particular article contains a summary and brief synthesis of these main emergent themes and discussions from the ICE 2018 course. In addition, we also provide suggestions on teaching the next generation of ICE scientists, especially during unprecedented global situations.

## Introduction

### Historical background of the insect chemical ecology PhD course series

Since the discovery of insect pheromones and the role of chemicals in plant–insect interactions, insect chemical ecology (ICE) has developed into a distinct research discipline being practiced at universities and other research organizations worldwide. Moreover, basic research in ICE has led to practical applications that contribute significantly to the control of agricultural or blood-feeding pests. High-quality education of PhD students in the field of ICE is of utmost importance for the discipline to grow and advance for future scientific discoveries as well as practical implementation. Recognizing the need for training and international networking of chemical ecologists, Bill Hansson and Ylva Hillbur initiated the first international ICE PhD course in 2005 at the Swedish University of Agriculture (SLU) involving teachers from Alnarp and Lund University, as well as many international lecturers. The interest of students and their positive response led to follow-up courses in 2007 and 2009 at Alnarp. In 2009, it was decided to rotate the location of the ICE courses and increase its frequency (from biennial to annual) to better accommodate the needs of international PhD students. As a result of this, a 2010 issue of the course was held at the Department of Entomology, Pennsylvania State University (PSU) and a subsequent one in 2012 at Max Planck Institute (MPI) for Chemical Ecology (CE) in Jena, Germany (where Bill Hansson had become the director and head of the Department of Neuroethology), merging resources and competence from three research institutions studying the chemical ecology of insects. With Teun Dekker at SLU (2009, 2011, 2015), Tom Baker at PSU (2010, 2014, 2017) and the Neuroethology group members (2012, 2016) at MPI, the course continued being arranged in turns by the three host institutions strongly promoting teacher and student exchange across borders. After the 2018 course, which is the subject of the current report, the International Centre of Insect Physiology and Ecology (ICIPE) in Nairobi, Kenya, joined the consortium of organizing institutions and arranged the first ICE PhD course in Africa in 2019, illustrating the establishment and development of the course as an internationally recognized platform for education and exchange of students, junior and senior chemical ecologists.

## Multitrophic interactions and plant defenses

Plants, insects, and microbes are part of terrestrial ecosystems. Trophic interactions, such as the consumption of plant material by herbivorous insects, are key factors that lead to physiological and morphological adaptations in coexisting organisms. For example, plants have evolved multiple defense mechanisms against herbivory, and insects likewise evolve countermeasures against plant defenses (Mello and Silva-Filho 2002). Following this line, a classical focus of ICE research has been the study of chemical defenses (allelochemicals) produced by plants in response to attack by herbivorous insects (Fraenkel 1959; Wittstock and Gershenzon 2002; van Dam 2012), whereas the functional role of specialized metabolites in direct defense is well established (reviewed in Howe and Schaller 2008), during the last few decades, an expansion of research interests to other chemical cues that mediate interactions between plants and insects culminated in the discovery of emissions of herbivore-induced plant volatiles (HIPVs) (Rhoades 1983). During the ICE PhD course, several contributing lecturers presented their own exploration into the interactions between a variety of crop species and different taxa of insect herbivores, natural enemies, plant-parasitic nematodes, and microbes.

**Fig. 1:**
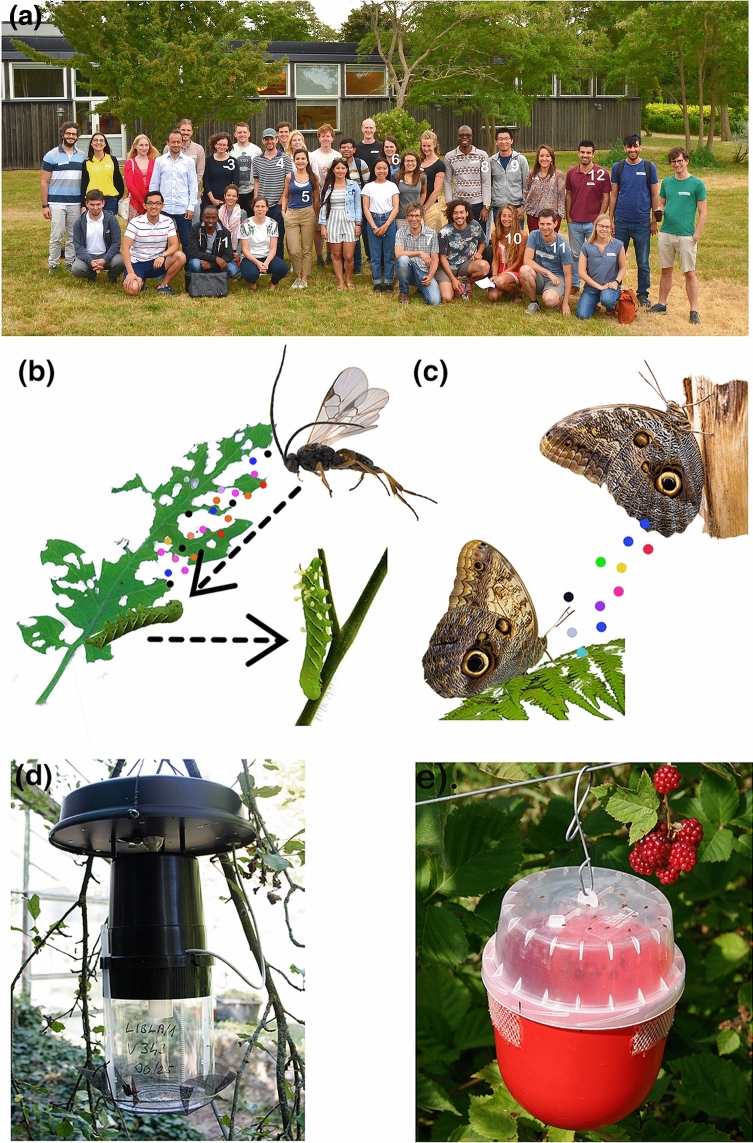
**a** Insect chemical ecology PhD course participants and organizers at the Swedish University of Agricultural Sciences (SLU), Alnarp Sweden. Names of the participants who authored this forum paper are numbered in white from left to right: (1). Crispus M. Mbaluto; (2). Magdolna O. Szelényi; (3). Anna L. Erdei; (4). William B. Walker III; (5). Gabriela Caballero-Vidal; (6). Alexandra G. Duffy; (7). Paul G. Becher; (8). Pascal M. Ayelo; (9). Siyang Xia; (10). Anaїs K. Tallon; (11). Urban Spitaler; (12). Gonçalo A. Duarte. **b** tomato leaf damaged by the caterpillar Manduca sexta and the release of herbivore-induced plant volatile (HIPVs- the coloured dots) to attract a natural enemy in the genus Cotesia. The natural enemy oviposit on the caterpillar, larvae develop inside the caterpillar and with time white cocoon emerge on the surface of the caterpilar. **c** female moth release pheromones (in coloured dots) to attract a male moth, **d** a field trap, and **e** a commercial trap for monitoring *Drosophila suzukii*. Source of pictures: panel (**b**) by Crispus M. Mbaluto, **c** obtained from Unsplash deposited by Paul Macalan and Hayley Maxwell (**d**) by Magdolna O. Szelényi, and (**e**) by Urban Spitaler. The figure artwork was done by Crispus M. Mbaluto

The release of HIPVs is involved in the indirect plant defense response that principally recruits natural enemies (i.e., predators and parasitoids) to the attacking insect(s) Fig. 1b (Turlings et al. 1990; Turlings and Wäckers 2004; Mumm and Dicke 2010). In a review paper by Aljbory and Chen (2018), 24 species belonging to 12 families of predators and 34 species belonging to 10 families of parasitoids had been reported as being attracted to volatiles emitted from plants infested by aboveground (AG) insects. Herbivore-induced cues allow the natural enemies to find and attack potential hosts, and in return, the plant is relieved of damage (McCormick et al. 2012). For example, in laboratory and field bioassays, several species of wasp in the genus *Cotesia* were found to be attracted to cabbage plants (*Brassica oleracea* var. *alba* L.) damaged by larvae of the diamondback moth (*Plutella xylostella* Linnaeus) and cabbage white butterfly (*Pieris rapae* L. Pieridae) (Poelman et al. 2009; Girling et al. 2011). Aside from the attraction of natural enemies to insect herbivores, it has been established that volatile compounds also serve as signals for herbivorous insects in terms of mating site and host plant selection, oviposition and feeding behavior, informing insects of plants infested by con- and heterospecifics, and induction of insect aggregation to overcome host plant defenses (Carrasco et al. 2015). The release of HIPVs and the attraction of natural enemies to insects do not only occur AG, but belowground (BG) dwellers also play critical roles in shaping biotic interactions within an ecosystem. A few examples presented in the AG-BG context showed that in roots, HIPVs are produced, and they can effectively recruit natural enemies to root-feeding insect herbivores in a comparable manner to AG systems (Rasmann et al. 2005; Degenhardt et al. 2009; Kivimäenpää et al. 2012; Ali et al. 2013). Another aspect to consider is plant–plant communication. HIPVs can act as rapid and potent aerial priming agents that prepare systemic tissues of the same plant, and neighboring plants for incoming attacks (Holopainen and Blande 2013). For example, herbivore-induced indole triggers an increase in the production of defensive volatiles in neighboring maize plants as priming agents (Erb et al. 2015; Li and Blande 2017).

Although these studies demonstrate the potential for the exploitation of volatile compounds from basic to applied research, more studies are needed to decipher in-depth the concept of blends and their meaning for insect behaviors. Furthermore, future research also needs to focus on the effectiveness of the recruited natural enemies (i.e., predators and parasitoids) in controlling specific insect pests, as well as the concentrations and active distances of emitted volatile blends.

Besides plant–insect interactions, the role of microbes in mediating a variety of biotic interactions is a growing research field in chemical ecology. Studies in the last decade have delved into complex microbial assemblages, i.e., microbiomes (for instance, bacteria, fungi, endophytes, floral microbes, etc.) that associate and interact with different plant species and their organs (Vorholt 2012; Hardoim et al. 2015; Compant et al. 2019). These interactions are ubiquitous and can vary between being beneficial, neutral, or pathogenic (Pineda et al. 2010, 2013; Martínez-Medina et al. 2017; Grunseich et al. 2019). Both beneficial and pathogenic plant microbes play important roles in modulating plant phenotype and cause wide-ranging effects on herbivore behavior and performance (Grunseich et al. 2019; Arif et al. 2020). For example, there is increasing evidence that soil-inhabiting microbes alter direct and indirect chemical defenses in plants, and this influences insect behaviors such as oviposition and foraging (Kempel et al. 2009, 2010; Pineda et al. 2010, 2013; Martínez-Medina et al. 2017; Bell et al. 2020). Elsewhere, recent studies have led to the discovery that nectar-inhabiting microbes change the composition of VOCs in host plant flowers and consequently influence the foraging behavior and survival of visiting insects such as herbivores, pollinators, and natural enemies (Beck and Vannette 2017; Rering et al. 2018; Klaps et al. 2020). For example, nectar inoculated by the yeasts, *Metschnikowia gruessii*, and *M. reukaufii*, attracts the parasitoid *Aphidius ervi* (Hymenoptera: Braconidae) via the production of volatile compounds (Sobhy et al. 2018).

Plants indirectly interact with the beneficial and symbiotic as well as pathogenic microbes that coexist with insects. A diverse community of microbes live within herbivorous insects and enter the plant during insect feeding. It has also been shown that insect-associated microbes can directly induce or suppress plant defenses after an herbivorous attack (Chung et al. 2013; Wang et al. 2017; Chen et al. 2020). Another perspective of insect-microbe associations suggests that microbes contribute to insect chemical defenses against potential predators and pathogens. For example, a symbiotic bacterium that is harbored by a tenebrionid beetle produces toxoflavin and caryoynencin, which protect the beetle’s eggs from pathogens (Flórez et al. 2017). Yet, another perspective of insect–microbe interactions suggests that microbes attract insects through the emission of volatiles to facilitate their dispersal (Christiaens et al. 2014; Becher et al. 2018). Other functions linked to microbes that need further examination include enabling insects to digest plant material, as well as mediating insect adaptation to the abiotic environment.

Abiotic factors, mainly the changes in global temperatures and differences in absorbed nitrogen (N) levels from soil, alter critical ecological processes and thereby have significant consequences on multitrophic plant-mediated interactions. An increase in temperature can change the direct defenses such as primary and secondary metabolites. For example, in vineyards, an increase in temperature slows down plant defense through reduced production of anthocyanins and amino acids, but an increase in tannins in the grapes, thus making the plants more susceptible to insect damage (Wu et al. 2019). Similarly, in several plant species, an increase in temperature triggers the release of higher amounts of volatiles, for instance, terpenoids and phenolics (Kleist et al. 2012; Holopainen et al. 2018). On the other hand, an increase in temperatures can accelerate insect metabolism and hence promote more feeding and herbivory (Havko et al. 2019). Based on the above examples on the impact of temperature on the plant’s chemistry and insect metabolism, it can be expected that more voracious arthropods pest populations will emerge (Havko et al. 2019). However, more studies are needed to provide details on how variations in temperature affect insect population dynamics and levels of plant damage or herbivory. The availability of nutrients such as N is an essential factor determining secondary chemistry within plants, including *Arabidopsis thaliana* and tomato plants (Hoffland et al. 1999; Stewart et al. 2001). The changes in N-based metabolites in plants influence insect herbivores as well as their natural enemies. For example, a high C/N ratio resulted in elevated *α*-tomatine concentrations in tomato plants, and at the same time, the plants became more susceptible to pathogens (Hoffland et al. 1999). Moreover, high N content can positively impact the abundance of natural enemies (Liman et al. 2017). Whether the attraction of natural enemies could be as a result of variations in C/N ratios and hence the emission of volatiles remains ambiguous. For omnivorous natural enemies, their high abundance on N-rich plants is attributed to the fact that their nutritional needs might be primarily met by the N-rich host plant (Liman et al. 2017). As a consequence, they may be less predatory. To this end, although only changes in temperature and N levels were discussed, future predictions highlight a plethora of new challenges that can only be deciphered via understanding how plants adapt to changing environments. Collectively, the body of studies discussed above demonstrates substantial progress in uncovering and understanding multitrophic interactions. However, the conceptual and empirical link between physiological changes, defense induction, and ecological impacts on insect populations persists at the forefront.

## Insect chemical communication: signaling, sensing, processing, and behavior among insects

Insects face different complex and heterogeneous environments and encounter a wide range of chemical odors. Their chemosensory systems facilitate the detection of chemicals odors in their surroundings, for recognition of potential mates (Fig. 1c), prey, oviposition sites, food sources, and to avoid enemies or harm. Sensing of chemical stimuli involves peripheral detection systems for compound recognition and central systems for processing to ultimately translate the chemical information into behavior (Leal 2013; Grabe and Sachse 2018).

The past 20 years has seen a steady increase in our understanding of insect gustation and olfaction. This began with the discovery of odorant receptors (ORs) (Vosshall et al. 1999), gustatory receptors (GRs) (Clyne et al. 2000), and later the ionotropic receptors (IRs) (Benton et al. 2009) as the primary chemoreceptor proteins housed in dendritic membranes of the olfactory sensory neurons (OSN) (Montagné et al. 2015). Among the three receptor families, the ORs—primarily in the genetic model organism, *Drosophila melanogaster*—remain the most widely studied in terms of function (Dobritsa et al. 2003; Hallem et al. 2004; Mathew et al. 2013). Data produced using the model system have been instrumental in explaining evolutionary biology across other insect species. For example, *Drosophila* antennae has been deployed as heterologous expression systems for the functional characterization of ORs from the noctuid moth *Spodoptera littoralis* in an empty neuron system (Dobritsa et al. 2003) or knock-in Or67dGAL4 system (Kurtovic et al. 2007). This has allowed the identification of ORs that are responsive to components of the female-produced pheromone blend (Montagné et al. 2012; Bastin-Héline et al. 2019), as well as those that are responsive to host plant volatiles (de Fouchier et al. 2017). Collectively, these findings have enabled the understanding of the evolution and adaptation of these receptors over time and across different populations or species. It has also opened up new ways for insect pest control (Venthur and Zhou 2018).

A growing number of studies have linked the evolution of chemosensory gene families such as ORs to insect adaptation to new ecological niches. According to the birth-and-death model of gene family evolution, chemosensory genes are gained via genomic duplication or lost via genomic deletion; (Vieira et al. 2007; Ramasamy et al. 2016; Missbach et al. 2020). The genes duplicates may remain in the genome for a long time, and whether they end up losing or gaining novel ecological functions is dependent on whether and to what extent combinations of drift and selection affect fitness. As an example, a high birth-and-death rate of ORs in *Drosophila suzukii* (Matsumura) resulted in reduced affinity for volatiles produced during fermentation and gained an affinity for short-chain esters found in fresh fruits substrate, which could be considered as an ecological adaptation to a specific niche (Ramasamy et al. 2016).

Some individual receptors detect specific odorants that are associated with crucial ecological adaptive roles. The evolution of the OR56a lineage in *Drosophila* flies is documented to confer identification and avoidance of unsuitable feeding and breeding sites (Stensmyr et al. 2012), while OR19a responds to signals from suitable substrates for oviposition (Dweck et al. 2013). The contribution to speciation via changes in OR affinity for species-specific odors is another insufficiently understood aspect in insect olfaction. A recent study on *D. melanogaster* flies isolated from an ancestral sub-Saharan woodland habitat revealed that volatiles from marula fruit induces species-specific host and oviposition site selection by activating Or22a or Or19a, respectively (Mansourian et al. 2018). Interestingly, most *Drosophila* species from southern Africa carry a specific allele at the Or22a/Or22b locus and are more sensitive to the marula ester ethyl isovalerate compared to the European *D. melanogaster* (Mansourian et al. 2018).

Investigations on odor coding, processing, and perception provide a global picture on the design of olfactory circuits that underlie the integration of behaviorally relevant olfactory information. A fundamental principle is that an odorant can activate a specific group of receptors (Carey and Carlson 2011). On the other hand, it is recognized that receptors which act as ‘generalist’ might also respond to overlapping groups of odorants, while ‘specialist’ responds to unitary or small sets of odorants (Carey and Carlson 2011). Thus, receptors include both broadly and narrowly tuned receptors consistent with dual information processing models of ‘labeled-line’ and ‘combinatorial across-fiber coding’ (Andersson et al. 2015). The insect sensory system seems to have evolved to function much like a spam filter, gating only signals that are necessary and sufficient for survival. For example, a study to unravel the unique affinity of *D. sechellia’s* for toxic morinda fruits revealed that the olfactory circuitry underwent several shifts at peripheral and central levels culminating in increased sensitivity and attraction to this particular fruit (Dekker et al. 2006). More studies are needed to elucidate how the olfactory repertoire in non-model insects might change during evolution and adaptation to new ecological niches.

Whereas much of our knowledge on insect olfaction stems from studies using the *Drosophila* model or some moth species, additional functions of chemosensation have been found in hymenopterans such as ants and bees. Studies on the desert ant (*Cataglyphis fortis* (Forel)) show the development of a well-structured olfactory memory that enables the ant to remember important olfactory landmarks. Further, the ants utilize the acquired knowledge to pinpoint their nest entrance in spatial scale (Steck et al. 2009). Similar to findings in ants, studies show that bees quickly learn to associate with important odors in their lives, and the ability to do so is faster and more reliable than their ability to learn visual cues (Sandoz et al. 2007; Wright and Schiestl 2009; Arenas and Farina 2012; Chakroborty et al. 2015).

In general, the field remains open for more discoveries due to the overwhelming number of insect species and the webs of multitrophic interactions between them and their ever-changing environments. High-throughput approaches such as genomics and transcriptomics based on next-generation sequencing are helping in the discovery and characterization of highly diverse chemoreception proteins and gene families (Montagné et al. 2015; Tian et al. 2018; Mitchell et al. 2020). The structural resolution of the insect OR coreceptor protein shines a light towards a better understanding of how ORs might interact with their odorant ligands (Butterwick et al. 2018; Zufall and Domingos 2018). Incorporating molecular engineering approaches outside the ‘model system’ can also provide immense new and relevant insights in chemical ecology. For instance, the Clustered Regularly Interspaced Short Palindromic Repeats/Cas9 (CRISPR/Cas9) genome editing system has been demonstrated as a highly efficient approach to study olfactory gene functions in a noctuid pest (Koutroumpa et al. 2016), as well as in other insects, including locusts and ants, demonstrating a broad relevance of this technique for chemical ecology research (Li et al. 2016; Trible et al. 2017; Yan et al. 2017; Fandino et al. 2019).

## Insect vectors of diseases

Vector-borne infections account for 17% of the human global infectious disease burden (National Academy of Science Engineering and Medicine, 2016). In recent years, studies on insects that vector disease-causing pathogens, such as ticks (Estrada-Peña et al. 2013), sand-flies (Alkan et al. 2013), triatomine bugs (Lazzari et al. 2013), and mosquitoes (Leal et al. 2017), have become an important branch of chemical ecology. This branch of chemical ecology provides opportunities to study and discover novel avenues for sustainable and integrative management of these disease-vectoring insects.

Here, we focus on mosquitoes, which have gained scientific attention from diverse research areas, including medical entomology, genomics, and chemical ecology, and were also a central theme in the ICE PhD course. Mosquitoes are globally distributed dipterans, and some species are of medical importance, especially those belonging to the family Culicidae (Tandina et al. 2018). Immediately after eclosion, both males and females feed on nectar or honeydew of plants to get sugars and other nutrients and that provide enough nourishment to live (Barredo and DeGennaro 2020). The females, however, need to produce eggs, which require proteins that they get from the blood of vertebrates. While taking a blood meal, these females, if already infected with the disease-causing pathogens, could transmit the pathogens to their host (i.e., humans or other vertebrates) (WHO 2014; Barredo and DeGennaro 2020). In order to combat mosquitoes, chemical ecologists have shifted in their conceptual thinking to unravel how mosquitoes recognize and discriminate resources during plant-seeking, host preference, and oviposition sites selection (Ignell and Hill 2020). These mosquito-behaviors are at least, if not mainly, mediated by chemosensation, and we briefly discuss them as holding promise for the development of new tools for surveillance and control of the disease-spreading mosquito species.

Amidst the vast diversity of plants in the environment, mosquitoes face the challenge of locating and discriminating plants with useful resources. Although mosquitoes also rely on vision and taste to find plants, olfaction is relied upon to detect important VOCs and decode them to locate nectar in distant plants (Barredo and DeGennaro 2020). Indeed, several studies agreed that mosquitoes rely on volatile chemical signatures, including terpenes, benzenoids, and aldehydes, to assess the suitability of a plant as a potential resource (Healy and Jepson 1988; Manda et al. 2007; Nikbakhtzadeh et al. 2014; Nyasembe et al. 2018). Although these studies demonstrate the importance of a single volatile compound in mediating plant-seeking behavior, the concept of blend recognition and how chemical codes regulate such behavior has been recently acknowledged (Ignell and Hill 2020).

Besides feeding on plant resources, mosquitoes use vertebrate blood as a nutritional resource for reproduction (Briegel 1990; Omondi et al. 2019). Most medically important species have been identified to express strong and inherent host selection behavior. Following this line, it has been further suggested that such behavior might have evolved in parallel with parasite-host evolution (Takken and Verhulst 2013). Chemical ecology research on the human-biting mosquitoes (e.g., *Aedes aegypti*, *Anopheles gambiae*) shows that both extrinsic and intrinsic factors drive host selection and preference. As reviewed by Takken and Verhulst (2013), extrinsic factors, including the presence of skin microbes (Ghaninia et al. 2008; Verhulst et al. 2010, 2011), host gender and age (Gallagher et al. 2008), and infection status of an individual (Robinson et al. 2018) shape the human body odor makeup that influences mosquito behavior. For instance, studies on human-emitted odor show preference of female mosquitoes to specific VOCs, including acetone, 1-octen-3-ol, and carboxylic acid (reviewed by Ignell and Hill 2020). Although laboratory and field assays show some level of success in using these and other volatiles to attract mosquitoes, it is still a challenge to define the ecologically relevant ratios and rate of release for these VOCs and their roles in mediating host attractiveness.

To further understand the mechanisms underlying host-preference behavior, the role of intrinsic factors such as variation in and expression of specific chemoreceptor proteins involved in host preference has been investigated (Omondi et al. 2015; Matthews et al. 2016; Taparia et al. 2017; Tallon et al. 2019). Indeed, studies demonstrate that specific ORs are expressed during blood-seeking behavior. For example, variations in the transcriptomes of the ancestral animal-biting ‘forest’ and human-biting ‘domestic’ forms of *Aedes aegypti* commonly found in tropical regions suggest that mosquitoes can distinguish between volatile cues emanating from humans and other vertebrates (McBride et al. 2014). The domestic form exhibits an increase in the expression and ligand sensitivity of the *Aaeg*Or4 gene to sulcatone (a human body sweat odor), which could explain the evolution of preference and specialization for human odors (McBride et al. 2014; McBride 2016). In light of the development of molecular tools, including genetically encoded calcium indicator (GCaMP) to visualize neuronal activity (Tian et al. 2009), and CRISPR/Cas9 genome editing (Kistler et al. 2015), it is possible to directly explore the genetics of insect disease vectors and molecular architecture of olfactory signaling. These technological advances give great hope for investigating the integration of genetic and neural changes by central brain circuits relevant to host selection and other behaviors (Kistler et al. 2015). With in-depth knowledge of how mosquitoes encode odors at the peripheral and central nervous systems, it will be a breakthrough to develop measures that simultaneously target different species that transmit different diseases.

The search for oviposition sites by gravid female mosquitoes after a successful blood meal is critical for the reproductive success of an individual and the population dynamics of the species. Gravid mosquitoes have to locate and recognize a suitable habitat (i.e., in terms of nutrition and void of natural enemies and competitors) to oviposit. Several factors, including water vapor and VOCs, guide the gravid mosquito in making choices at increasingly fine scales (Afify and Galizia 2015; Wondwosen et al. 2016, 2017). Understanding the interaction between mosquitoes and these factors during oviposition site selection is a crucial avenue currently being investigated for insights on integrated insect control. Water vapor serves as a signal to a gravid mosquito on the presence of a water body (Matthews et al. 2019). Aside from the water itself, odors emanating from vegetations, including grasses, have been associated with a suitable larval habitat (Asmare et al. 2017a; Wondwosen et al. 2018). Soil microbes associated with vegetation near water bodies have been recently reported to provide attractants that regulate selection oviposition sites (Takken and Knols 1999; Ignell and Hill 2020). Efforts to identify VOCs in these habitats report monoterpenes and aldehydes as the prominent compounds present in the odor blends that attract the mosquitoes to these habitats and also stimulate oviposition (Wondwosen et al. 2016, 2017, 2018). Furthermore, based on interpreting the presence and ratios of these VOCs, it has been demonstrated that mosquitoes can also establish a hierarchical habitat preference within diverse vegetation (Asmare et al. 2017a). VOCs originating directly from water bodies have been ecologically associated with the mosquito’s acceptance of the potential habitat. Following this line, it has been suggested that acceptable habitats might be signaled by the presence of conspecifics (Seenivasagan et al. 2009; Wachira et al. 2010) and are less crowded, rich in nutrients, and without the presence of predators and competition (Vonesh and Blaustein 2010). The role of VOCs in mediating these ovipositional responses is under investigation. For instance, the VOCs originating from habitats with the presence of potential predators so far remain largely unidentified. Whereas investigation on the potential VOCs is essential, there is also a need to understand how environmental cues such as carbon (C) and nitrogen (N) ratio in a substrate and movement through differing landscapes influence the breeding success of mosquitoes (Asmare et al. 2017b). That aside, there are still other open ecological questions regarding how climatic changes and CO_2_ levels could explain shifts in oviposition site selection as well as geographic range expansion and host preference over time.

Overall, the data in mosquito chemical ecology demonstrate that the recognition and discrimination of blends is vital for the selection of plants, hosts, and oviposition sites. Further, the olfactory system is adapted to decoding blends of VOCs both qualitatively and quantitatively in order for the mosquito to make decision in increasingly fine scales. However, there is a need to decipher more on blend recognition, from mechanistic to ecological scales.

## Incorporating insect chemical ecology in diversified agroecosystems

The endeavor of agriculture requires sustainable crop protection methods for predictable and economical food production. For several decades, conventional pesticides have served the world well; however, for sustainable pest management, there is a need to replace these external applications with environmentally friendly crop protection approaches (Fig. 1d, e). To this end, ICE provides an in-depth understanding of the origin, functions, and significance of natural chemicals (hereafter referred to as semiochemicals) that mediate multitrophic interactions between organisms such as plants, insect herbivores, and their natural enemies.

Semiochemicals provide additional control options to conventional pesticides (El-Shafie and Faleiro 2017; Mauchline et al. 2018). Sex pheromones of some Diptera, Lepidoptera, and Coleoptera species have been successfully used in pest control. As an example, the application of sex pheromones as mating disruptors and lures successfully reduced swede midge in Brassicaceae (Hillbur et al. 2005; Samietz et al. 2012). Similarly, sex pheromones from heliothine moths and codling moth (*Cydia pomonella*-Linnaeus) have been used successfully to disrupt mate location (Witzgall et al. 2010). In forestry, aggregation pheromones of bark beetles (order; Coleoptera) are frequently used in trap-out methods (Gillette and Munson 2007). Pheromones and HIPVs have also been applied in crop fields to attract predators and parasitoids and thus provide an additional novel alternative to exploit them via biological control (Vosteen et al. 2016; Turlings and Erb 2018). For example, field application of the aphid alarm pheromone (E)-*β*-farnesene (Eβf) recruited parasitoids and predators of aphids (Vosteen et al. 2016). Moreover, the incorporation of methyl salicylate, a well-known HIPV, in slow or controlled-release field dispensers attracts predators of spider mites in grapes and hops agroecosystems (James and Price 2004) and natural enemies to aphids in soybean (Mallinger et al. 2011). In another study, the release of methyl salicylate in cranberry (*Vaccinium macrocarpon*) plants was found to increase the number of visitations and predation of sentinel eggs by adult lady beetle, adult hoverflies, and predatory mites (Salamanca et al. 2019). Besides their use in pest management, pheromones are also reliably used as monitoring tools in biodiversity conservation. This has been demonstrated using sex pheromones of the saproxylic beetles, which successfully help to monitor the beetles in both managed and natural forest ecosystems (Musa et al. 2013). This highlights that insect pheromones are robust indicators of biodiversity and can revolutionize conservation efforts in diverse insect groups.

The role of pheromones, HIPVs, and other semiochemicals can be exploited to develop an effective and sustainable multidisciplinary approach for plant protection. A key example is the push–pull system in which the principal or target plant is protected by a combination of two components: a source of repellent signals that reduce pest colonization and development ‘push’ and a source of attractant cues ‘pull’ which include trap plants grown on the perimeter of the main crop to attract the pest. The incorporation of semiochemicals in the context of ‘push–pull’ cropping systems offers excellent potential for pest control in cases where existent inputs provide insufficient control (Khan et al. 2010; Pickett et al. 2014). Along the same line, the recent advances in biochemistry and molecular genetics enable critical advancement in the engineering of plants that produce a chemical compound that offers the potential for managing insects of crop plants (Degenhardt et al. 2003; Bruce et al. 2015). For example, genes that regulate pheromone biosynthesis in insects were introduced in *Nicotiana benthamiana* leaves for the production of pheromonal components detectable to hundreds of moth species (Ding et al. 2014). Similarly, with the advancement in biochemistry, molecular genetics, and genetic engineering techniques, a hexaploidy variety of wheat *Triticum aestivum* cv. Cadenza (Poaceae) was engineered in the laboratory to release Eβf, the alarm pheromone for many pest aphids (Yu et al. 2012; Bruce et al. 2015). The release of Eβf demonstrated intrinsic activity against three pest aphid species (a grain aphid: *Sitobian avenae* Fabricius, bird cherry-oat aphid: *Rhopalosiphum padi* Linneaus, and rose-grain aphid: *Metopolophium dirhodum* Walker) and increased the foraging by *Aphidius ervi* (Ervi), the natural enemy of these aphids (Bruce et al. 2015). Whereas these laboratory-based studies demonstrate the considerable potential for pest control, field trials are critical to evaluate the concepts further. In addition, more studies using different crop species are needed.

Although the use of semiochemicals is a promising pest management strategy, there are challenges for their implementation in the real world. The registration of semiochemicals for biological controls can be a lengthy process and is sometimes not granted. Collaboration between research, extension officers, farmers, and semiochemical supply industries facilitates the successful exploitation of semiochemicals. In many countries, such collaborations are either underdeveloped or lacking; we therefore urge collaborative initiatives, as they provide avenues to exploit the economic dynamics of targeted semiochemicals. As an example, for trials in South Tyrol and Trento regions in Italy, collaboration between farmers, researchers, and the industrial sector confirms the successful implementation of semiochemicals (e.g., mating disruptors) in pest management strategies against the codling moth in apple orchards and *Lobesia botrana* (Denis & Schiffermüller) in vineyards (Anfora et al. 2009; Ioriatti et al. 2011). Another example of a successful pest management approach, courtesy of a collaboration between stakeholders, stems from the popular ‘push–pull’ technology developed in sub-Saharan Africa. Through effective multilevel partnerships between national agricultural research and extension systems, non-governmental organization, and other stakeholders, the ‘push–pull’ technology has been widely disseminated to reach one million farm households and effectively reduce populations of pest in the Lepidoptera family, e.g., the stemborer moths: *Busseola fusca* Füller (Noctuidae) and *Chilo partellus* Swinhoe (Crambidae), and fall armyworm larvae, *Spodoptera frugiperda* (J.E. Smith) in maize crops (Khan et al. 2014; Midega et al. 2018).

## Organizing and participating in insect chemical ecology course during a global pandemic

In 2020, the outbreak and rapid spread of novel coronavirus disease (COVID-19) broadly affected almost all aspects of human endeavor, including health, economics, social interactions, and education, among others. Education is a critical sector in modern society and has been primarily achieved best by teaching and training in a face-to-face environment. Following these lines, the ICE course for PhD students in chemical ecology and closely related research had enjoyed a face-to-face format annually for over a decade, while rotating between research facilities spread across three continents. Each year, both students and the selected trainers (established and leading scientists in chemical ecology) would converge at one of the participating research facilities for the course activities. In 2020, the cycle was broken due to the unprecedented Covid-19 pandemic.

What options do the ICE organizers and trainers have in responding and teaching the next generation of ICE scientists during a global pandemic, where social distancing is mandatory? In general, the advancement in technology has enabled the shifting of various human interactions from predominantly in-person to predominantly virtual. Such an unprecedented shift from the classical meeting in a host city or laboratory to cyber interactions during the Covid-19 pandemic became a new way to continue scientific exchange in an unprecedented time and has presented an opportunity for accelerated implementation of digital teaching and training technologies, and ‘unprecedented opportunities’ to ensure students do not miss out. Other positive aspects of using these technologies are reduced overall costs (e.g., travel and accommodation) and the inclusion of more students that otherwise could not participate due to either limited course capacity or else limited financial resources. Here, we suggest some ideas on how the ICE course can be successfully conducted during unprecedented situations and going forward even during more normal times.

As pandemics such as Covid-19 demand restricted movement and social interactions, the first option to conduct ICE would be virtual-only attendance and teaching. This option offers real-time interactions over the internet. Several platforms for audio, videos, and chatting have been developed to enable interactive online learning experiences including lectures, discussion, and group training. Besides the virtual-only meeting, we suggest embracing the hybrid type of meeting where only students and trainers residing near the participating research facility can be allowed to participate in in-person and laboratory demonstrations. At the same time, those who would need to travel from far can follow the lectures and discussions virtually. The last option is pre-recording lectures and laboratory demonstrations. Here, the trainers can pre-record their lectures and demonstrations and have them streamed or played for the students and followed by a question-and-answer session with the particular lecturers. With this approach, copyright can be an issue. Therefore, we suggest the use of the non-downloadable format of the records or the lectures get played from a central platform and only for a limited time. While ICE courses are not just about teaching only, the students and trainers also get a chance to interact socially and talk about experiences with career progression. In all of the above-proposed methods of organizing and teaching the next ICE courses, we suggest the continued inclusion of social engagement activities even under a virtual setting, where both the trainers and students can interact and exchange from a social perspective, which is critical for students as early career scientists.

## Concluding remarks

Insects possess an inimitable capability to generate and recognize chemical cues and interact with a wide range of organisms in their environment. While these processes are only partly understood, the body of studies presented in this article demonstrates steady progress in understanding ICE and closely related fields. Indeed, ICE is a continuously growing and promising field of research where insights gained when studying these natural ecological interactions trigger new questions and sustainable contributions to the field and applications in agriculture and vector-borne disease control. Advancements in methods and instrumentations are accelerating manipulation of the molecular architecture and chemical events underlying signal emission, olfaction, and behavior. This is deepening our knowledge and helping to answer broader chemoecological and evolutionary questions, especially those linked to agriculture and disease vectors. Because ICE is an interdisciplinary field, the collaboration between professionals and early career scientists working in behavioral ecology, electrophysiology, analytical chemistry, molecular biology, data-driven science, medical entomology, and agroecology, among other disciplines, is vital. The ICE PhD course with its yearly organization rotating between SLU Alnarp in Sweden, PSU in the USA, MPI for Chemical ecology in Jena, Germany, and since 2019, ICIPE in Nairobi, Kenya, with the involvement of lecturers from other leading ICE research institutions, is a successful avenue for students to gain insights into the cutting edge of ICE research. Moreover, the course offers an opportunity to build bridges between disciplines, inspire new questions, and establish professional networks between participating students and lecturing scientists. We challenge the early career ICE scientists to follow and develop the present knowledge beyond their known borders.

## Data Availability

Data sharing is not applicable to this article as no datasets were generated or analyzed during the current study. Code(s) sharing is/are not applicable to this article as there were no code or codes generated.
